# TRANSLATION, CROSS-CULTURAL ADAPTATION AND PRELIMINARY EVALUATION OF THE BRAZILIAN VERSIONOF THE PAIN CATASTROPHIZING SCALE-PARENTS

**DOI:** 10.1590/1984-0462/;2018;36;4;00014

**Published:** 2018

**Authors:** Julianna Amaral Cavalcante, Karolline Alves Viana, Paulo Sucasas Costa, Luciane Rezende Costa

**Affiliations:** a Centro Universitário Unievangélica, Anápolis, GO, Brasil.; B Universidade Federal de Goiás, Goiânia, GO, Brasil.

**Keywords:** Pain, Catastrophization, Child behavior, Validation studies, Dor, Catastrofização, Comportamento infantil, Estudos de validação

## Abstract

**Objective:**

In Brazil, there is no scale to assess parental catastrophizing about their child’s pain. This study aimed to translate and cross-culturally adapt the Pain Catastrophizing Scale-Parents to the Brazilian Portuguese language, as well as to preliminarily evaluate its psychometric properties among parents/guardians of children with and without a toothache.

**Methods:**

A cross-sectional study was conducted with 237 parents/other relatives of 237 children. Across-cultural adaptation of the scale into Brazilian Portuguese was carried out according to the universalistic approach. To assess the reliability and validity of the scale, parents/other relatives reported on the child’s toothache and filled out the Brazilian versions of the Pain Catastrophizing Scale-Parents and the Dental Discomfort Questionnaire.

**Results:**

There was semantic equivalence with the original version after minor modifications. TheCronbach’s alpha for the 13 items of the scale was 0.83, and the respective test-retest intraclass correlation coefficients ranged from 0.63 to 0.97. The scores obtained from the Pain Catastrophizing Scale-Parents and the Dental Discomfort Questionnaire had a low correlation (rho=0.25; p<0.001). Thetotal score of the Pain Catastrophizing Scale-Parents differed significantly (p<0.001) in children with a toothache at night (median: 3.0, 25-75 percentile: 25.0-35.5) compared to those who did not have a toothache at night (25.5; 20.0-31.0).

**Conclusions:**

The Brazilian version of the Pain Catastrophizing Scale-Parents was acceptable in this preliminary evaluation and can be used in Brazilian clinical and research practice.

## RESUMO

**Objetivo:**

No Brasil, não há escala que avalie a catastrofização dos pais sobre a dor das crianças. O objetivo deste estudo foi traduzir e adaptar transculturalmente o *Pain Catastrophizing Scale-Parents* para a língua portuguesa do Brasil e avaliar preliminarmente as propriedades psicométricas de pais/outros parentes de crianças com e sem dor de dente.

**Métodos:**

Foi realizado um estudo transversal com 237 pais/outros parentes de 237 crianças. A adaptação transcultural da escala para o português brasileiro foi feita conforme a abordagem universalista. Para avaliar a confiabilidade e a validade da escala, os pais/outros parentes fizeram um relato sobre a dor de dente da criança e preencheram as versões brasileiras da Escala de Catastrofização da Dor-Pais e o Questionário de Desconforto Dentário.

**Resultados:**

Houve equivalência semântica com a versão original após pequenas modificações. O alfa de Cronbach para os 13 itens da escala foi 0,83 e os respectivos coeficientes de correlação intraclasse do teste-reteste variaram de 0,63 a 0,97. Os escores obtidos na Escala de Catastrofização da Dor-Pais e no Questionário de Desconforto Dentário apresentaram baixa correlação (rho=0,25; p<0,001). O escore total da Escala de Catastrofização da Dor-Pais diferiu significativamente (p<0,001) em crianças com dor de dente à noite (mediana: 30,0; percentil 25-75: 25,0-35,5) quando comparado com o daquelas sem dor de dente (25,5; 20,0-31,0).

**Conclusões:**

A versão brasileira da Escala de Catastrofização da Dor-Pais apresentou características aceitáveis nesta avaliação preliminar e pode ser utilizada no Brasil tanto na prática clínica quanto em pesquisas.

## INTRODUCTION

Catastrophic thoughts can be defined as mental processes that are negative and exaggerated, and occur as a response to an unpleasant experience.^1^ These catastrophic thoughts increase the intensity of pain,^2^ the feeling of physical disability,^3^ stress,^4^ and inadequate response to treatment.^5^ Ithas been demonstrated that a psychological mechanism (pain catastrophizing) influences the biological phenomenon of the increased pain experience found for unpredictable stimuli.^6^ Asystematic review has indicated that pain catastrophizing is related to areas of the brain that are involved in the processing of and attention to pain, reduction of pain inhibition, and other cognitive-affective aspects, such as emotions and motor activity.^7^

Pain catastrophizing, biased information processing regarding a threat, reflects the person’s tendency to integrate pain-related cognitive-affective factors into a holistic pain experience, ultimately modulating the pain experience.^8^ Theway in which social background influences pain and the individual’s behavior before a painful experience has largely been disregarded. Thus, in this context, it is crucial to identify the elements that contribute to disability, and to provide tools to measure them.^9^

There is a positive association between levels of pain catastrophizing by parents and by their children. Moreover, a family may have a specific cognitive style to deal with the pain associated with the child’s responses, when he or she feels pain.^10^ Inthis regard, the Pain Catastrophizing Scale-Parents(PCS-P) was developed in 2006 to evaluate the response patterns of parental catastrophizing about pain in their children.

The PCS-P is internationally recognized, but, to the best of our knowledge, has not been adapted for use in Brazil. Several studies have confirmed the clinical utility and psychometric properties of these measures. However, to date, in Brazil there is no culturally sensitive instrument that is available to evaluate children’s behavior in situations of chronic pain nor one that focuses on family establishment as a reinforce of pain perception. Another version of the Pain Catastrophizing Scale(PCS) has been validated in Brazil,^11^ ^,^ ^12^ but it is directed toward adult patients with specific chronic pain conditions, not toward parents/other relatives of children in pain, justifying our study. Thus, this study aimed to translate and cross-culturally adapt the PCS-P to the Brazilian Portuguese language, as well as to preliminarily evaluate psychometric properties among parents/other relatives of children with and without a toothache.

## METHOD

**Figure 1:**
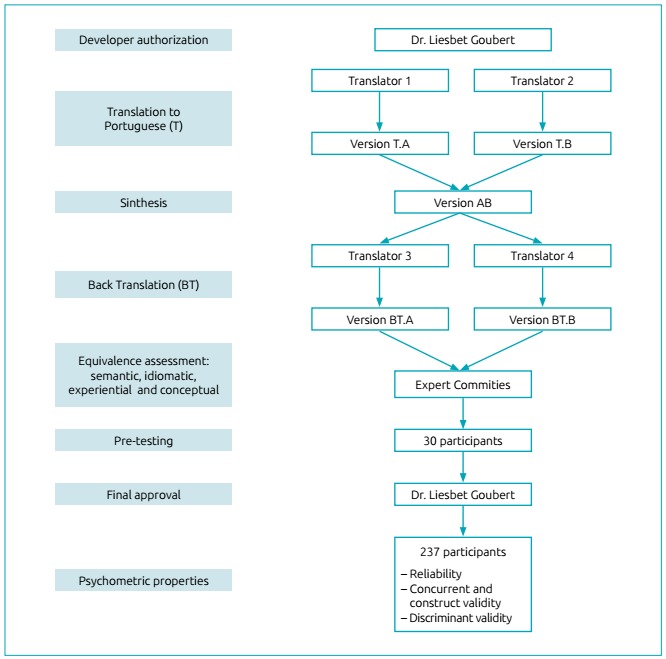
Flowchart depicting the process of cross-cultural adaptation and assessment of psychometric properties of the Brazilian version of the Pain Catastrophizing Scale-Parents.

This study followed the Declaration of Helsinki’s principles,^13^ the recommendations from Resolution 466/2012 of the National Council of Health from Brazil Ministry of Health,^14^ and was approved by the Research Ethics Board at the Universidade Federal de Goiás, Goiânia (GO), Brazil (protocol no.363/2010). Allparticipants (professionals who participated in the adaptation phase as well as the children’s parents/other relatives) were individually informed about the investigation and asked to sign a consent form if they found it appropriate. Thestudy was based on the universalist approach to cross-cultural instrument adaptation,^15^ which understands that the meaning of an instrument’s items should be adjusted for each culture, even if there is an underlying universal concept. Itwas performed in multiple standardized phases (Figure1)^16^ ^,^ ^17^ ^,18^ that can be merged into two stages:

1. translation and cross-cultural adaptation;
2. preliminary evaluation of psychometric properties.

The PCS-P is a self-administered questionnaire that assesses the extent of parents’ catastrophizing thoughts, feelings, and behavior when their children are in pain.^9^ Itwas developed due to the need to relate the extent to which parents catastrophize the pain of their children with the impact on the parents’ wellbeing and the child’s behavior. Thus, it investigates whether parental catastrophic thinking about pain explains the difficulty of children in dealing with a painful situation, the anxiety of these children and the same intensity of pain.^9^ Furthermore, the PCS-P assesses whether there is a significant positive correlation between parental behavior and how their children experience and express pain.^10^ Itconsists of 13items with five possible responses, which are rated on a five-point Likert-type scale: not at all(0), mildly(1), moderately(2), severely(3), and extremely(4). Items are grouped into three subscales: helplessness (items1, 2, 3, 4, 5, and 12), magnification (items6, 7, and 13), and rumination (items8, 9, 10, and 11). The total score on the scale could range from 0 (zero) to 52 (corresponding to the multiplication of 13 items by a score of 4).

The developer of the PCS-P authorized its translation and cross-cultural adaptation to Brazilian Portuguese, and it was carried out in accordance with previously published guidelines.^16^ ^,^ ^17^ ^,^ ^18^ Two bilingual translators (T1 and T2), whose native language is Brazilian Portuguese, translated the PCS-P separately and produced two independent written translations. T1 was aware of the concepts that were being examined in the questionnaire in order to provide equivalence from a clinical perspective. T2 was not informed about the concepts to be investigated, in order to offer a translation that better reflected the language used by the majority of the population (a lay translator). T2 highlighted ambiguous meanings in the original questionnaire. The translations were compared and discrepancies were solved after a discussion among T1, T2, and an observer. Finally, a common translation was achieved.

Then, two translators who were born in an English-speaking country and were literate in the language performed a back-translation of the Portuguese version of the PCS-P into English. Thetwo translators did not have training in health sciences and were unaware of the instrument’s concepts. Theback translation was performed in order to ensure that the translated version reflected the same content as the original item. Theresulting version of this stage was discussed by a group of experts. Theexpert committee was formed by a professor with expertise in survey questionnaires, three health professionals (one pediatrician and two pediatric dentists), a professional with a degree in the Portuguese language, one translator, and one back-translator, with the goal of consolidating all of the versions of the questionnaire and to develop the pre-test version of the Brazilian Portuguese PCS-P questionnaire. Theexpert committee made decisions for the equivalence between the original PCS-P version and the target version in four areas: semantic, idiomatic, experiential, and conceptual.^16^

The pre-test version of the questionnaire was administered to a group of 30people who answered the questionnaire with the guidance of the researcher. They were then interviewed to see if they had understood the meaning of the questions and had responded appropriately, in order to ensure that the adapted version maintained its equivalence in the applied condition. Researchers qualitatively analyzed the pre-test version and sent a report to the PCS-P developer, who approved the translation and cross-cultural adaptation process and the Brazilian-Portuguese version of the PCS-P, after a few suggestions.

The Brazilian-Portuguese PCS-P psychometric properties were tested in a sample of 237 parents/other relatives of children aged six years old or younger. The sample was non-probabilistic, and the sample size was based on the study that developed the original scale.^9^ The parents/other relatives were recruited in the reception area of five dental clinics of public and private practices in two large cities in central Brazil. Inclusion criteria were children aged less than or equal to six years old that had a mother, father, or other relative that was available to answer the questionnaires. Participants would be excluded if they did not fully respond to the instruments.

In the reception area of the dental offices, one of the two trained researchers individually interviewed each parent/other relative using the Brazilian-Portuguese PCS-P and the Brazilian Dental Discomfort Questionnaire(DDQ-B).^18^ ^,^ ^19^ The DDQ-B was used as an observational measure of the child’s dental pain, analyzing the concurrent and construct validity properties of the PCS-P, given that we intended to check whether PCS-P was able to be used in assessments of patients with dental pain/discomfort. The DDQ-B is comprised of two parts: the first directly asks the caregivers if they think the child has a toothache, including a toothache at night (while sleeping); the second part is comprised of 12items concerning the child’s behaviors regarding a toothache, with a score varying from0 (nopain) to 24(the worst possible pain). Toanalyze the test-retest stability, 20parents/other relatives answered the PCS-P again after 14days.

Reliability was assessed by stability (test-retest) and internal consistency (homogeneity) tests. Test-retest reliability was determined by calculating the intraclass correlation coefficient (ICC). Degree of reliability was estimated based on the following ICC values: ≤0.40=poor, 0.41 to 0.60=moderate, 0.61 to 0.80=good, 0.81 to 1.00=excellent.^20^

The homogeneity of the PCS-P, considered as a whole and with regard to factors, was measured using Cronbach’s alpha, which is an analysis that captures the extent of agreement between all possible sets of responses. Values ≥0.70 were considered acceptable.^21^

Concurrent and construct validity were analyzed by investigating the association between the scores obtained in the PCS-P and the DDQ-B, to order to observe if the PCS-P would measure different aspects of pain related to dental pain/discomfort. Apositive correlation by the Spearman correlation test was expected.

The discriminant validity of the PCS-P was determined by comparing the PCS-P scores and the occurrence of a toothache at night (while sleeping). TheMann-Whitney test was used to investigate whether or not parents/other relatives in charge of children with a toothache at night would have more catastrophic thoughts than those responsible for children without this symptom, because pain at night can negatively impact the whole family. Statistical analyses were performed using IBM SPSS Statistics v.19, with the significance level set at p-value<0.05.

## RESULTS

**Figure 2:**
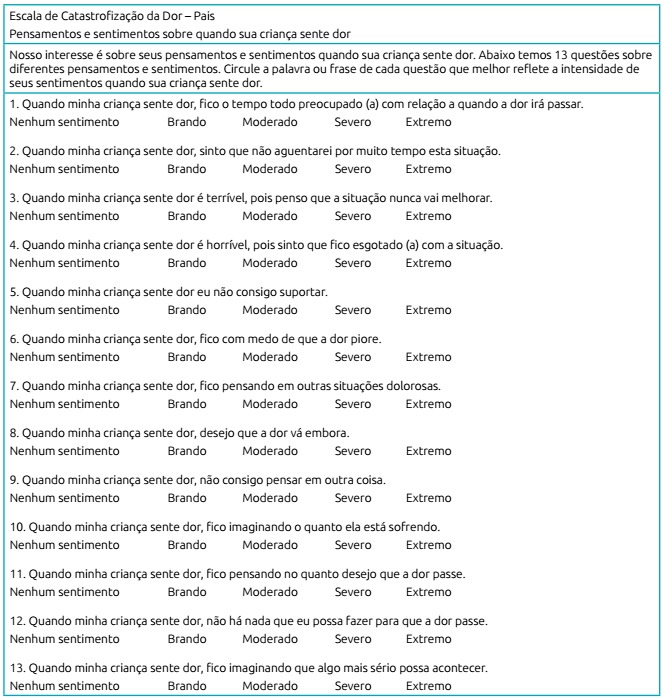
The Brazilian-Portuguese version of the Pain Catastrophizing Scale-Parents (Escala de Catastrofização da Dor-Pais”.

In relation to the translation and cross-cultural adaptation, a few issues were found and solved throughout the diverse steps of the process (Table1). Some changes were required following the pre-test phase, since survey participants questioned if they should give answers just regarding dental pain or with regard to any pain symptoms. Also, they tended to interpret the answer choices as frequency (always, sometimes, rarely, never), not intensity. After considering the comments of everyone involved in this adaptation process, and performing appropriate changes, the Brazilian-Portuguese PCS-P was proposed. Hereinafter it is referred to as the *Escala de Catastrofização da Dor-Pais* (ECD-P) (Figure2).

**Table 1:** Issues occurring during the translation and cross-cultural adaptation steps of the Pain Catastrophizing Scale-Parents (PCS-P).

Issue*	Solution
Questionnaire instructions and items: the word “child” was translated by T1 as “son” and by T2 as “child”.	It was standardized to translate as child, because the questionnaire can be answered by a child’s caregiver and not only by the parents.
Questionnaire instructions: the expression “is in pain” was suggested to be changed to “feel pain” by the expert committee.	Accepted, because it better fits colloquial Brazilian Portuguese.
The word “please”: T1 did not translate or keep the word “please”, explaining that, in Portuguese, the questions are used in imperative format.	For cultural reasons, it was decided against the use of “please”.
Answer options: T1 suggested changing the intensity responses to frequency responses.	To maintain the semantic equivalence and, in the future, if we conduct another study, we will change the response to frequency (never, ever) or agreement (partially agree, strongly agree, etc.)
Answer options: T1 suggested “no feeling, mild feeling, moderate feeling, severe feeling, extreme feeling”, whereas T2 kept the original version options “not at all, mildly, moderately, severely, extremely”.	To change to the “feeling” options. However Prof L. Goubert advised not to keep the word “feeling” because the questionnaire mainly assesses “thoughts”.
T1 removed the personal pronoun “I” in all items and T2 kept it.	To remove the personal pronoun “I” from all of the items to better comply with colloquial Portuguese
Item 5, “When my child is in pain, I cannot stand it anymore”: T1 eliminated the word anymore and T2 kept it.	To eliminate the word “anymore” order to avoid redundancy in Portuguese

*T1: Translator 1; T2: Translator 2

The participants included 175mothers (73.8%), 28fathers (11.8%), and 34 other relatives (14.3%), who were accompanying 237children aged 1.1 to 6.0years old (mean=4.1, standard deviation=1.3). Among the children, 51.9% were boys. The parents/other relatives reported that 79children (33.3%) did not have a toothache, 109 (46.0%) had it sometimes, 40 (16.9%) had it often, and 9 (3.8%) did not know; 29.1% reported that their child had a toothache at night.

The overall score for the ECD-P followed a non-normal distribution (Kolmogorov-Smirnov, p=0.03) had a median of 26.0 (25-75 percentile: 21.0-32.0), and the frequency of responses varied along the items of the instrument (Table2). Considering each item of the scale, the test-retest ICCs ranged from 0.63 to 0.97 (Table2). The internal consistency (Cronbach’s alpha) value for the 13items of the ECD-P was 0.83. The analysis of the item-total correlation indicated that there would be no improvement in this value if any item were removed. Byanalyzing the factors separately, it was found that the Cronbach’s alpha was 0.76 (helplessness), 0.70 (rumination), and 0.62 (magnification).

**Table 2:** Relative frequencies for the responses to the Brazilian version of the Pain Catastrophizing Scale-Parents, and test-retest stability.

Subscales Items (When my child is in pain…)	Frequency of answers* (%)					ICC
	1	2	3	4	5	
Helplessness						
1. I worry all the time about whether the pain will end.	0.4	18.1	11.4	51.1	19.0	0.82
2. I feel I can’t go on like this much longer.	16.9	30.8	16.5	30.0	5.9	0.96
3. It’s terrible and I think it’s never going to get better.	42.2	21.9	11.4	19.4	5.1	0.95
4. It’s awful and I feel that it overwhelms me.	26.2	27.8	9.7	29.5	6.8	0.93
5. I can’t stand it anymore.	34.2	27.8	16.5	16.0	5.5	0.83
12. There is nothing I can do to stop the pain.	40.9	21.5	13.1	19.0	5.5	0.90
Magnification						
6. I become afraid that the pain will get worse.	1.7	27.8	11.0	40.5	19.0	0.63
7. I keep thinking of other painful events.	27.8	33.3	8.9	24.1	5.9	0.95
13. I wonder whether something serious may happen.	13.9	30.0	8.9	38.4	8.9	0.97
Rumination						
8. I want the pain to go away.	0	5.5	4.6	51.9	38.0	0.91
9. I can’t keep it out of my mind.	8.0	21.9	11.0	40.5	18.6	0.90
10. I keep thinking about how much he/she is suffering.	1.7	10.1	11.4	56.1	20.7	0.92
11. I keep thinking about how much I want the pain to stop.	4.2	4.6	4.2	61.2	25.7	0.85

*1= Not at all; 2= Mildly; 3= Moderately; 4= Severely; 5= Extremely. ICC= Intraclass correlation coefficient.

There was a low positive correlation between the scores obtained in the overall ECD-P and the DDQ-B (rho=0.25, p<0.001), as well as in the ECD-P factors and the DDQ-B: rumination (rho=0.26, p<0.001), helplessness (rho=0.17, p=0.01), and magnification (rho=0.15, p=0.03). Children with a toothache at night had a higher overall ECD-P score (median: 30.0, 25-75 percentile: 25.0-35.5) than those without toothache at night (25.5; 20.0-31.0) (p<0.001, Mann-Whitney test).

## DISCUSSION

This study found that the ECD-P showed semantic equivalence with the original version, after minor adjustments were made throughout the systematic and universalist process of cross-cultural adaptation. Furthermore, the ECD-P presented acceptable psychometric properties studied herein, which allows it to be employed in future investigations focusing on children’s health care. After all, interventions aiming to change parents’ attitudes towards their children’s pain behaviors should assess parents’ catastrophic thoughts about the child’s pain.^22^

The ECD-P showed acceptable internal consistency, which means there was consistency across item responses. It should be highlighted that the overall Cronbach’s alpha for ECD-P was greater than 0.80, which is the value generally recommended for psychometric scales.^23^ Thus, from this perspective, the ECD-P is suitable for both group analysis and the interpretation of individual scores. Itcould be argued that this result should be viewed with caution, given that the determination of a single Cronbach’s alpha for the scale of 13items as a whole is not theoretically correct, because, by definition, Cronbach’s alpha indicates the correlation between the items that measure a single construct, and the ECD-P is a scale in three dimensions. However, when considering the Cronbach’s alpha for the subscales, values were around 0.70, and the magnification (alpha=0.62) and helplessness (alpha=0.76) coefficients were close to those showed in the study that developed the original PCS focused on adult catastrophizing (alpha=0.60 and 0.79, respectively).^1^

We chose to assess psychometric properties of the ECD-P based on a dental pain model mainly because: the prevalence of Brazilian preschoolers with toothaches is high (22.0%),^24^ toothaches in preschoolers have a negative impact on on the families’ quality of life ^25^ ^,^ ^26^ and is associated with work absenteeism by parents.^27^ Thus, parents might have catastrophic thoughts about their children’s toothache due to the harmful effects on their lives. Furthermore, a few reports in the field of dentistry have highlighted the influence of catastrophic thinking on dental pain in adults.^6^ ^,^ ^8^ ^,^ ^28^

Interestingly, DDQ-B and ECD-P showed satisfactory construct validity in this study, which was expected. Indeed, the low correlation coefficient indicates that the ECD-P scores explain 25% of the variance in DDQ-B. Although the DDQ-B and the ECD-P assess different concepts, i.e., toothache and parental catastrophizing about their child’s pain, one might expect that they could be slightly correlated, considering that the suffering of children with a toothache could evoke catastrophic thoughts in their parents. Thereby the ECD-P would be appropriate as a component of a set of instruments aiming to assess patients with pain resulting from cavities. Similarly, in a previous study, the Brazilian version of the PCS showed positive and significant correlations with other pain-related aspects, such as pain intensity, pain interference, and patient mood.^11^

Accordingly, the ECD-P demonstrated the capability to differentiate between children with decayed teeth, that is, a higher total score of catastrophic thoughts was found among parents/other relatives of children with one or more decayed teeth. This probably occurs because children with tooth decay can suffer from a toothache, as the tissue damage related to dental cavities often causes pain,^19^ ^,^ ^25^ so parents/other relatives catastrophize the pain of the children. Itshould be mentioned that the relationship between cavities and toothache was also revealed in the present study.

This study has some limitations. First, although we attempted to include a large sample size, our sample was non-probabilistic. Thus, it was not representative of the Brazilian population and did not allow for a confirmatory factor analysis. Second, we did not apply other measures of family stress that could help with the construct validity of the ECD-P. Third, social characteristics of the respondents (e.g. formal education and family income) were not collected and could have influenced in the results. Nonetheless, this study adds to the literature, and our results support the use of the ECD-P in research in different healthcare settings. Understanding parents’ reactions to their children’s pain offers a perspective regarding a family’s pain experiences and improves diagnoses and treatment.^29^

In summary, the ECD-P (the Brazilian version of the PCS-P) showed conceptual equivalence of items and semantics with the original scale, and also presented proper reliability, reproducibility, and discriminant and construct properties. Future studies could extensively investigate the factor structure of the ECD-P in different Brazilian populations, verify the effects that may occur in changing the response to frequency (never, ever) or agreement (partially agree, strongly agree, etc.), and seek relationships between pain in children and caregivers’ catastrophic thoughts.
